# Cutting-Edge Research on Physical Fitness Profile in Soccer Players

**DOI:** 10.3390/sports13110372

**Published:** 2025-10-29

**Authors:** Yiannis Michailidis

**Affiliations:** Laboratory of Evaluation of Human Biological Performance, Department of Physical Education and Sports Sciences, Aristotle University of Thessaloniki, 54124 Thessaloniki, Greece; ioannimd@phed.auth.gr; Tel.: +30-231-099-2248

Soccer is one of the most popular sports in the world, with millions of athletes engaged in it. The sport evolves over time, and players continuously develop their physical, technical, and tactical skills in order to meet its increasing demands. Technological advances have enabled a detailed investigation of the physical fitness profile required for high performance, as well as the quantification of many factors related to training load.

However, despite these advances, in several areas of the game, the scientific literature remains limited or findings are inconclusive. Much of the research focuses on isolated performance indicators without examining their interactions, either with one another or in relation to competitive conditions. Furthermore, most studies involve elite male soccer players, with women, youth, and amateur athletes being underrepresented. In addition, there is still considerable scope for research into training protocols aimed at improving/maximizing performance, preventing injuries, and supporting return-to-play following injury.

This Special Issue of *Sports*, entitled *Cutting-Edge Research on Physical Fitness Profile in Soccer Players*, addresses some of these gaps by compiling scientific contributions on modern soccer. Specifically, the 15 articles included examine physical capacities, performance monitoring strategies, match analysis, recovery, and injury prevention frameworks. These studies also involve male and female players of different ages and competitive levels. Both experimental and review articles are included.

Within the Special Issue, several articles focus on tracking external load using GPS technology, both during matches and training sessions. Such studies aim to clarify the demands of different playing positions and inform the structuring of more effective training microcycles. Other studies investigate specific intervention programs and the role of feedback in performance and learning. Furthermore, bibliometric and review papers address topics such as decision-making, injury-prevention markers, and return-to-play criteria. Additional contributions examine the effects of contextual factors (e.g., temperature) on performance and explore the relationships between running performance and physical capacities. Taken together, this Special Issue offers a multidimensional and updated perspective on how profiling the physical fitness of soccer players can support evidence-based practice in the sport.

Looking ahead, long-term monitoring across different levels of play and both sexes is needed to clarify position-specific demands. Moreover, further research should focus on the use of artificial intelligence for more efficient training design, as well as on big data analytics for predicting performance and injury risk. A key goal of future studies must be the integration of findings into applied practice.

